# Sinusitis among Patients Undergoing CT Scan of Paranasal Sinuses in a Tertiary Care Centre: A Descriptive Cross-sectional Study

**DOI:** 10.31729/jnma.7595

**Published:** 2022-10-31

**Authors:** Abhushan Siddhi Tuladhar, Anish Bhattarai, Sneha Bimali, Bibek Pokharel, Sunil Pradhan, Amit Shrestha, Riwaz Acharya

**Affiliations:** 1 Department of Radiology, Nepal Medical College and Teaching Hospital, Jorpati, Kathmandu, Nepal; 2 Nepal Medical College and Teaching Hospital, Jorpati, Kathmandu, Nepal

**Keywords:** *computed tomography*, *paranasal sinuses*, *sinusitis*

## Abstract

**Introduction:**

Computed tomography imaging provides detailed information about the paranasal sinuses and is now well established as an alternative to standard radiographs in evaluating patients with sinusitis. Sinusitis can have dangerous complications which can even lead to death if not diagnosed and treated on time. This study aimed to find the prevalence of sinusitis among the patients undergoing Computed Tomography scan of paranasal sinuses in a tertiary care centre.

**Methods:**

A descriptive cross-sectional study was conducted in the Department of Radiology at a tertiary care hospital from 1 October 2021 to 30 April 2022 after taking ethical approval from the Institutional Review Committee (Reference Number: 023-078/079). The patients undergoing Computed Tomography paranasal sinuses and meeting the eligibility criteria were enrolled for the study after taking informed consent. Convenience sampling was done. Point estimate and 95% Confidence Interval were calculated.

**Results:**

Among 113 patients, 109 (96.46%) (93.05-99.87, 95% Confidence Interval) patients had sinusitis. The most common subtype was found to be acute sinusitis in 63 (57.79%) individuals.

**Conclusions:**

The prevalence of sinusitis was higher than in other studies done in similar settings.

## INTRODUCTION

Sinusitis is defined as inflammation of the mucous membrane of paranasal sinuses or underlying bone.^1^ Based on the duration of symptoms, it can be divided into acute rhinosinusitis, subacute rhinosinusitis, and chronic rhinosinusitis. While imaging is not usually recommended for patients with acute uncomplicated rhinosinusitis, computed tomography (CT) scan of the sinuses without contrast is the imaging of choice for patients with recurrent acute or chronic sinusitis.^2^

While conventional plain radiography is limited to demonstrate maxillary and frontal sinus diseases, they provide limited views of the anterior ethmoidal cells, the upper two-thirds of the nasal cavity, and the frontal recess.^3^ CT imaging provides detailed information of the paranasal sinuses and is now well established as an alternative to standard radiographs.^4,5^

This study aimed to find out the prevalence of sinusitis among the patients undergoing CT scan of paranasal sinuses in a tertiary care centre.

## METHODS

A descriptive cross-sectional study was conducted in the Department of Radiology at Nepal Medical College and Teaching Hospital for 7 months from 1 October 2021 to 30 April 2022 after taking ethical approval from the Institutional Review Committee (Reference number: 023-078/079) from the same institution. All the patients above 12 years undergoing CT paranasal sinuses (PNS) were enrolled for the study after taking informed consent. Patients with past history of facial trauma and patients who had undergone facial or nasal surgeries in the past were excluded from the study. Convenience sampling was done. The sample size was calculated by using the formula


$$\begin{array}{ll} \text{n} & ={\text{Z}}^{2}\times \frac{\text{p}\times \text{q}}{{\text{e}}^{\text{2}}}\\ & =1.96^{2}\times \frac{0.50\times 0.50}{0.10^{2}}\\ & =96 \end{array}$$

Where,

n= minimum required sample size
Z= 1.96 at 95% Confidence Interval (CI)
p= prevalence taken as 50% for maximum sample size calculation
q= 1-p
e= margin of error, 10%

Hence, the minimum required sample size is 96. A sample size of 113 was collected.

Sinusitis is defined as an inflammation of the mucous membrane of the paranasal sinuses or their underlying bone. Mucosal thickening, air-fluid levels, and ostio-meatal complex blockade are the important CT scan findings in sinusitis.^1^ A proforma was developed to identify the presence of sinusitis based on symptoms. Patients were also asked about the duration of symptoms, history of treatment, and presence or absence of symptoms at the time of the CT scan.

CT scan was performed with a series of millimeter 0.5 mm slices using Toshiba, Aquilion 64 slice CT scanner and analyzed by using Vitrea software. To avoid observer bias, the study was done by a single observer. The axial, coronal, and sagittal planes were used for evaluation. The thickness of the maxillary mucosa was measured on plain CT sections and its inflammation and obstruction were noted in the same.

The collected data were coded and entered into Microsoft Excel. Data was entered and analyzed in IBM SPSS 21.0. Point estimate and 95% CI were calculated.

## RESULTS

Among 113 patients undergoing CT scan of paranasal sinuses, 109 (96.46%) (93.05-99.87, 95% CI) were found to have sinusitis. Acute and chronic sinusitis was found in 63 (57.79%) and 38 (34.86%) patients respectively (Table 1).

**Table 1:** Types of sinusitis (n= 109).

Chronicity	n (%)
Acute sinusitis	63 (57.79)
Chronic sinusitis	38 (34.86)
Fungal sinusitis	8 (7.33)

CT scan showed mucosal thickening as the most common finding in both acute sinusitis 49 (44.95%) and chronic sinusitis 31 (28.44%) and the ostiomeatal complex pattern was found in 14 (12.84%) patients with chronic sinusitis (Table 2).

**Table 2:** CT scan findings (n= 109).

Findings	n (%)
**CT findings in acute sinusitis**	
Mucosal thickening	49 (44.95)
Ostiomeatal complex blockade	41 (37.61)
Mucoperiosteal reaction	28 (25.69)
Hypertrophied turbinates	19 (17.43)
Air fluid level	17 (15.60)
Hemosinus	3 (2.75)
Air bubbles within fluid	1 (0.92)
**CT findings in chronic sinusitis**	
Mucosal thickening	31 (28.44)
Ostiomeatal complex blockade	18 (16.51)
Polyps	12 (11.01)
Calcification	6 (5.50)
Hyperostosis of bone	5 (4.59)
Sinonasal polyposis	5 (4.59)
**Pattern of chronic sinusitis**	
Ostiomeatal complex pattern	14 (12.84)
Sporadic pattern	12 (11.01)
Sinonasal polyposis pattern	5 (4.59)
Sphenometal recess pattern	4 (3.67)
Infundibular pattern	3 (2.75)

Maxillary sinus was found to be involved in 60 (55.05%) with bilaterality in ethmoidal sinusitis in 28 (25.69%) and pansinusitis in 13 (11.92%) patients (Table 3).

**Table 3:** Distribution of sinusitis (n= 109).

Sinuses involved	n (%)
**Frontal**	39 (35.78)
Right	18 (16.51)
Left	10 (9.17)
Bilateral	11 (10.09)
**Maxillary**	60 (55.05)
Right	23 (21.10)
Left	16 (14.68)
Bilateral	21 (19.27)
**Sphenoidal**	27 (24.77)
Right	9 (8.26)
Left	11 (10.09)
Bilateral	7 (6.42)
**Ethmoidal**	
Bilateral	28 (25.69)
Left	5 (4.59)
Right	3 (2.75)
**Pansinusitis**	13 (11.92)

Out of total patients with sinusitis, 23 (21.10%) patients had developed complications, the mucocele was seen in 12 (11.01%). Studying other incidental coexisting pathological conditions of PNS, osteoma in 33 (30.27%) patients had the maximum count followed by solitary polyp/retention cyst in 32 (29.36%) patients and antrochoanal polyps in 27 (24.77%) patients (Table 4).

**Table 4:** Complications and other incidental pathologies in patients with sinusitis (n= 109).

Variable	n (%)
**Complications**	23 (21.10)
Mucocele	12 (11.01)
Periapical abscess	3 (2.75)
Orbital cellulitis	2 (1.83)
Brain abscess	1 (0.92)
Orbital abscess	1 (0.92)
Oroantral fistula	1 (0.92)
Subdural empyema	1 (0.92)
Subperiosteal abscess of orbit	1 (0.92)
Dural venous sinus thrombosis	1 (0.92)
**Incidental pathological conditions**	
Osteoma	33 (30.27)
Solitary polyp/retention cyst	32 (29.36)
Antrochoanal polyps	27 (24.77)
Concha bullosa	26 (23.85)
Fibrous dysplasia	3 (2.75)
Juvenile angiofibroma	2 (0.18)
Odontogenic cyst	2 (1.83)
Carcinoma of the maxillary sinus	2 (1.83)
Underdeveloped maxillary sinus	2 (1.83)
Inverted papilloma	1 (0.09)
Silent sinus syndrome	1 (0.92)
Mucocele/mucopyocele	7 (6.42)
Cherubism	1 (0.09)

The mean age of the patients was 40.67±14.2 years with female predominance 65 (59.63%). The male: female ratio was 1:1.5 (Table 5).

**Table 5:** Age and gender-wise distribution (n= 109).

Age groups (years)	Male n (%)	Female n (%)	Total n (%)
13-20	3 (2.75)	4 (3.66)	7 (6.42)
21-30	6 (5.50)	10 (9.17)	16 (14.67)
31-40	16 (14.67)	25 (22.94)	41 (37.61)
41-50	8 (7.33)	12 (11)	20 (18.35)
51-60	5 (4.58)	6 (5.50)	11 (10.09)
61-70	4 (3.66)	7 (6.42)	11 (10.09)
>71	2 (1.83)	1 (0.92)	3 (2.75)
Total	44 (40.36)	65 (59.63)	109 (100)

## DISCUSSION

Our study showed the prevalence of sinusitis to be 96.46% with 57.79% acute sinusitis being the most common subtype. Mucosal thickening was the most common CT finding and the most affected paranasal sinus was found to be the maxillary sinus (55.05%). Mucocele (11.01%) was the most common complication while osteoma (30.27%) was the most commonly associated incidental finding.

Among 113 symptomatic patients enrolled in the study, 109 patients were found to have sinusitis which shows about 96.5% prevalence which is higher than a previous study done in the US which was 81%, and another similar prospective study done in India which was 65%.^5,6^ Sinusitis is a common condition affecting almost all patients coming for a CT scan of the paranasal sinuses. Although less frequent in occurrence, fungal sinusitis is a distinct and important pathology that requires special attention. The use of CT scan not only helps identify the subtype, characteristics, and pattern of sinusitis but also aids in the detection of major complications involving the head and neck. The use of CT has also helped discover various incidental pathologies co-occurring along with sinusitis including carcinoma which aids in the early treatment of the disease.

Based on the duration of symptoms, sinusitis can be divided into 3 subtypes: acute rhinosinusitis (<4 weeks), subacute rhinosinusitis (4-12 weeks), and chronic rhinosinusitis (>12 weeks).^2^ Since inflammation of paranasal sinuses is almost always accompanied by inflammation of the nasal cavity, the term rhinosinusitis is sometimes preferred over sinusitis.^7^ The overall incidence was found to be 12% in the United States (US).^8^ The prevalence of chronic rhinosinusitis alone was found to be 15% in the United Kingdom (UK) and 6.4% in the US.^9,10^ Conventional plain radiography is limited to demonstrating maxillary and frontal sinus diseases, they provide limited views of the anterior ethmoidal cells, the upper two-thirds of the nasal cavity, and the frontal recess. CT scan, on the other hand, provides detailed information on the anatomy and pathophysiology of sinus disease of all paranasal sinuses.^3^

The most commonly involved age group in our study was 31-40 years (37.6%) similar to the previous crosssectional study done in India which was 28%.^6^ Diagnosis of rhinosinusitis cannot be made alone on the basis of symptom-based diagnostic guidelines as set forth by the Task Force on Rhinosinusitis (TFR).^11^ Hence, more emphasis is laid on the role of CT in detecting and characterizing different types of rhinosinusitis.

A study done in the US showed air-fluid levels as being more specific to acute sinusitis.^12^ Our studies showed similar results as well. Mucosal thickening was the most common CT finding in our study similar to a previous comparative study done in Spain which showed 54.9% of patients with mucosal thickening.^13^ The most common pattern of sinonasal inflammation for chronic sinusitis in our study was the ostiomeatal complex pattern which is different from the study done in the US which showed a predominance of the infundibular pattern (26%).^14^ Complications occurred in 21.1% of patients enrolled in our study with mucocele being the most common followed by orbital complications. This is in contrast to a retrospective study done in Ireland that showed 44.2% of patients presented with orbital complications.^15^

The sensitivity of ten selected coronal slices of limited CT scan in accurately diagnosing and identifying sinusitis was found to be as high as 92%.^16^ And with a complete set of multiplanar CT Scan images of paranasal sinuses, like in a study of ours, the sensitivity and accuracy of sinusitis are even higher. Hence, a CT scan is an essential tool in identifying sinonasal inflammatory diseases and their complications. When used early in the course, it can aid in diagnosing coexisting pathologies as well as provide appropriate treatment for early recovery of the patients.

Patients with facial trauma, and patients who had undergone facial or nasal surgeries were excluded from the study, as there was a possibility of misinterpretation as sinusitis. As the data was collected from one hospital only, and with a small sample size, the final result represents a part of the population and should not be generalized to represent the whole population.

## CONCLUSIONS

The prevalence of sinusitis was slightly higher as compared to other studies done in similar settings. However, the predominant findings in CT were similar to other studies. The major complications meanwhile were different in our study. Sinusitis is a disease of significant public health importance. CT scan remains a standard tool for providing appropriate details of sinonasal inflammation which aids in early diagnosis and treatment.
